# A dataset to facilitate automated workflow analysis

**DOI:** 10.1371/journal.pone.0211486

**Published:** 2019-02-07

**Authors:** Tony Allard, Paul Alvino, Leslie Shing, Allan Wollaber, Joseph Yuen

**Affiliations:** 1 Defence Science & Technology Group, Edinburgh SA, Australia; 2 MIT Lincoln Laboratory, Lexington MA, United States of America; Liverpool John Moores University, UNITED KINGDOM

## Abstract

Data sets that provide a ground truth to quantify the efficacy of automated algorithms are rare due to the time consuming and expensive, although highly valuable, task of manually annotating observations. These datasets exist for niche problems in developed fields such as Natural Language Processing (NLP) and Business Process Mining (BPM), however it is difficult to find a suitable dataset for use cases that span across multiple fields, such as the one described in this study. The lack of established ground truth maps between cyberspace and the human-interpretable, persona-driven tasks that occur therein, is one of the principal barriers preventing reliable, automated situation awareness of dynamically evolving events and the consequences of loss due to cybersecurity breaches. Automated workflow analysis—the machine-learning assisted identification of templates of repeated tasks—is the likely missing link between semantic descriptions of mission goals and observable events in cyberspace. We summarize our efforts to establish a ground truth for an email dataset pertaining to the operation of an open source software project. The ground truth defines semantic labels for each email and the arrangement of emails within a sequence that describe actions observed in the dataset. Identified sequences are then used to define template workflows that describe the possible tasks undertaken for a project and their business process model. We present the overall purpose of the dataset, the methodology for establishing a ground truth, and lessons learned from the effort. Finally, we report on the proposed use of the dataset for the workflow discovery problem, and its effect on system accuracy.

## 1 Introduction

The prevalence of Information and Communication Technology (ICT) and their function as a critical capability enabler now poses a risk for organizations should they become degraded, compromised, or inoperable [1]. In a military context, commanders want to develop risk management processes to protect their ICT capability enablers and provide *mission assurance*, where Mission Assurance (MA) is defined as “measures required to accomplish essential objectives of missions in a contested environment” [2].

To realize appropriate risk management, commanders must understand how their mission is enabled by ICT infrastructure and the impacts to MA should it become compromised, defective, or unavailable. However, it is challenging to map the infrastructure to the missions that they are supporting (and vice versa) without an up-to-date understanding of how infrastructure is being used to accomplish tasks and achieve mission goals. We contend that an understanding of task semantics is an important input to the commander’s comprehension of the mission relevant cyber terrain. We seek to associate individual tasks with the mission(s) they support and the cyber terrain they use. We define the artifact of such a process a *mission map*. The mission map reveals key entities in the battle space (e.g., missions, tasks, workflows, hardware, services, people, and data) that support the commander’s mission. It shows the semantic relationships (e.g. dependencies) between these entities, which help the commander to comprehend how these entities work together to achieve mission success and the impact to the mission outcomes from the loss or degradation of mission relevant cyber terrain.

In order for an organization to achieve its mission, it must execute one or more mission-critical tasks. For example: in an accounting organization, one of these tasks may be to prepare tax returns on behalf of a client; in a military organization, one of these tasks may involve resupplying forward operating bases. Regardless of the tasks being executed, we propose that many of them follow *workflows*, which may be thought of as a template for repeated tasks, and that parts of a workflow must be executed using resources within the cyber domain. It is our hypothesis that such workflows can be learned, at least in part, from network data sources, such as emails, chat logs, and event logs. The ability to automate workflow discovery algorithms is of particular interest since manual workflow extraction from data is time consuming, and the addition of automation may help improve the efficiency of workflow analysis techniques.

It is essential that we compare results produced at each step of any workflow analysis pipeline against those from a known ground truth dataset in order to adequately evaluate the algorithm’s performance. Additionally, any supervised natural language processing techniques and algorithms forming part of such a pipeline (for examples, see those described in [3, 4]) require labeled training data to be used. Current process discovery techniques attempt to mine and discover workflow models using knowledge obtained from event logs [5], and, recently, natural language descriptions of the models themselves [6]. However, these event logs (e.g. transaction logs for financial institutions and hospital logs) often lack the level of detail and frame of reference necessary to effectively correlate events to their high-level missions. We presume that event characterization from *natural language documents* may provide the necessary mission context that these event logs lack. These documents (e.g. emails, chat logs, or web content) contain unstructured event information that can be parsed using natural language processing (NLP) techniques to learn patterns or extract central themes from such unstructured text that could then be automatically regularized into event logs. These event logs, enriched with natural language context, can then be used as input for process discovery algorithms.

Ground-truth datasets do exist for areas of NLP. For example, teams have manually annotated subsets of WordNet [7, 8], a lexical database created in the Cognitive Science Laboratory of Princeton University that groups English words into sets of synonyms and their relationships among common synonym sets. These manually annotated datasets may be used to quantify the performance of word sense disambiguation techniques [9–11] as well as other NLP approaches. Similarly, various Business Processing Models [12–14] have ground truth sequences which allow evaluation of business process discovery techniques. However, the authors have yet to find a dataset with sufficient ground truth that combines natural language categorization with the extraction of repeating event sequences to identify workflows.

In this paper, we explain the method by which we created a ground truth for a publicly available email dataset extracted from the mailing list of an open source software project. Such a ground truth dataset supports the development of machine learning algorithms for workflow analysis. This serves the dual purpose of allowing future researchers in the area of workflow analysis to transparently understand the methodology that was used to establish the ground truth, including our novel presentation of mapping trace instances to workflows (described below), as well as providing a way to replicate the approach using an extension of our source data or any other source data.

The paper is structured as follows. Section 2 defines a formalism and methodology for workflow analysis. Section 3 describes our requirements for a validation dataset and our data selection process. Section 4 explains the method for constructing a ground truth dataset, and includes an explanation of the modified Delphi process used to construct the consensus-driven labeled dataset. Section 5 details the results attained at the various Delphi process meetings, and Section 6 follows with a discussion of these results. Related work is discussed in Section 7, and Section 8 concludes the paper and discusses future work.

## 2 Workflow analysis

Modern organizations often establish workflows to help control and monitor how their employees perform certain defined business functions or tasks (e.g., travel, leave approval, procurement, and recruitment). Having a defined workflow allows organizations the ability to complete tasks in a somewhat predictable and measurable way. It can also aid in associating an organization’s mission essential tasks to underlying resources used within the cyber terrain. Often it is difficult to determine whether defined workflows are actually followed; or if undefined, what actions and workflows are executed on an organizations cyber resources. Workflow analysis defines the systematic approach to identify and characterize tasks that are executed within an organization.

A workflow is defined by the set of actions involved to perform a task, and the logic constraints that govern how such actions may be executed with respect to each other.

**Definition 1 (Workflow).** A directed graph (digraph) *G*(*A*, *D*) where:

1. *A* = {*a*_1_, …, *a*_*n*_} is the set of actions executed within the workflow, that make up the graph vertices, and,
2. *D* = {(*a*_*i*_, *a*_*j*_), …, (*a*_*m*_, *a*_*n*_)} is the set of directed edges between actions, indicating how actions are temporally ordered or linked.

When a workflow is performed, a subset of actions are executed that follow a particular path defined by logic constraints. We term this a *workflow instance*. Not all actions are required to be present in an instance.

**Definition 2 (Workflow Instance).** A sequence of *actions* *a*_*i*_ ∈ *A* that are executed in order to complete a workflow, *τ*^*A*^ = (*a*_1_, …, *a*_*m*_).

When workflows are instantiated, they may follow different paths consisting of varying actions. These paths manifest themselves as a sequence of observed events, (*e*_1_, …, *e*_*n*_). We constrain our approach to datasets consisting of events described by natural language content such as email, chat, and web content. We term such events *natural language events*. In order to perform workflow analysis on the datasets under consideration, the semantics of each event, *e*_*i*_ ∈ *E*, must relate back to the actions in *a*_*j*_ ∈ *A*. It is possible that each event may describe more than one action within a workflow or actions in multiple workflows. We derive labels for each event, both directly from the natural language within the event (keyword label), and indirectly using an external frame of reference (metalabel). The process of metalabel enrichment with knowledge from external sources (e.g. semantic databases, dictionaries, etc.) may improve the accuracy of event characterization with respect to *A*.

**Definition 3 (Keyword Label).** A task-relevant word, *k*, taken directly from a natural language event that helps describe the nature of the event.

**Definition 4 (Metalabel).** A task-relevant word, *m*, not necessarily taken from the natural language event itself, that helps provide context of the event.

Each event *e*_*i*_ is described by the sets *K*_*i*_ and *M*_*i*_, where *K*_*i*_ is a set of keyword labels *K*_*i*_ = {*k*_1_, …, *k*_*n*_}, and *M*_*i*_ is a set of metalabels *M*_*i*_ = {*m*_1_, …, *m*_*n*_}. Events are then arranged into one or more event-sequences based their semantics and the context in which they are observed. We hypothesize that each sequence describes an instantiation of one or more workflows. We term such a sequence of events a *trace*, *τ*.

**Definition 5 (Trace).** A sequence of observable events *e*_*i*_ ∈ *E* that are executed in order to complete a workflow, *τ*^*E*^ = (*e*_1_, …, *e*_*m*_).

Each event must belong to at least one trace, even if that trace comprises only one event. Given a finite time window of observation, it is possible that only some events within a trace will be observed. Unobserved (missed) events of a trace may occur outside of the time window or may be overlooked due to imperfect observations. Traces that are not fully observed are called *partial traces*. If there are sufficient partial traces of the same workflow, then it is possible to learn a complete workflow from partial traces.

Putting all this together, given a data set of temporally ordered, natural language events *E*, workflow analysis can be performed by constructing functions (via human supervision or machine learning) that, build upon each other, to map:

1. natural language events *e*_*i*_ to sets of keywords *k*_*i*_, K:E→K,
2. events and keywords to metalabels, M:(E,K)→M,
3. events, keywords, and metalabels to event traces, $T:\left(E,K,M\right)\to \tau^{E}$,
4. event traces and metalabels to workflow instances $A:\left(\tau^{E},K,M\right)\to \tau^{A}$, and,
5. workflow instances to workflows, $G:\tau^{A}\to G\left(A,D\right)$.

The problem of workflow analysis, then, hinges on the reliable construction of the functions K, M, T, A, and G that use natural language events to produce keywords, traces, metalabels, workflow instances, and, ultimately, workflows. In this paper we describe the ways by which we manually create a consensus-driven, labeled dataset that will ultimately be used to test machine learning techniques for each step in the workflow analysis pipeline outlined above.

## 3 A validation dataset

Manually extracting workflows from a dataset is a resource intensive task, especially when there are no defined workflows a priori. Thus we seek to develop a process of automatic workflow extraction from natural language datasets, within the broader context of developing a mission map for MA. To empirically evaluate our techniques we require a dataset that has the following properties:

- *Natural language events*: the events in the dataset must contain at least one user-populated, natural language field critical for event characterization. Examples of such events include email, chat, wiki pages, and forum threads. Counterexamples are packet headers and host authentication logs.
- *Data availability*: the dataset must be fully accessible, without redaction/de-identification.
- *Contains Workflows*: the events within the dataset must follow one or more defined workflows.
- *Ground truth*: each observed event must be mapped to workflow actions, and each event trace defined and mapped to the workflow it represents.

After an extended search, no dataset existed that satisfied all of our desired requirements. The open source software project email archive from the Apache Software Foundation (ASF) was the best candidate, satisfying all requirements except the existence of a ground truth.

### 3.1 The Apache Software Foundation (ASF) email archives

All active projects under the ASF have a dedicated community responsible for managing the development and release of an Apache product such as Hadoop HDFS [15], ActiveMQ [16], and Camel [17]. In keeping to their philosophy of openness, all communication and updates regarding product development generally route through several mailing lists that are eventually published and available to the public to browse. Anyone across the globe can join a project-specific community as a user or developer. Participants engage in a prescribed process to report and review bugs, contribute code to a future release version, or join the committee that handles task allocation and release management. Additionally, many Apache projects use various software management tools such as Atlassian Jira [18] (issue tracking and management), Git [19] (distributed version control), and Internet Relay Chat (IRC) (instant messaging). Each of these services (except IRC) automatically sends emails to the project mailing lists to inform community members of other developers’ activities. The email corpus satisfies our requirements as follows:

- *Natural language features*: Both the subject line and email body contain natural language text.
- *Data availability*: The corpus contains emails spanning more than 10 years, and did not appear to have significant gaps or missing emails throughout this time period. Additionally, all text and content is open-source and seemed unmodified from the original version.
- *Contains Workflows*: The Apache project developer community provides a structured wiki that documents instructions on how issue resolution or code release should be performed. This indicates that workflows should exist within the dataset. JIRA and Git instances are used to manage the software development process, and consequently enforce workflows on certain processes.
- *Ground truth*: Currently, the mapping of events to their respective workflow action(s), or a series of events to a trace, is not available and would thus need to be constructed.

After evaluating each Apache project against our criteria we decided to use the Apache Camel dataset, because it contains multiple sources of data, is vertically aligned, and contains a number of JIRA issues addressed by the Apache Camel project community. Although the Apache Camel dataset consists of multi-source data, we focused our ground truth activity only on email, which is comprised of the following, publicly available mailing lists:

- *Dev*: Development of the project, code releases, project management, etc.
- *Users*: General discussion and support amongst users and developers.
- *Issues*: Bug reports, software testing, task assignment.
- *Commit*: Automated notifications sent by various ASF version control tools such as SVN, CVS, and Git.

### 3.2 Dataset selection

We next discuss our method of selecting a time window for our Apache Camel email dataset. There are over 500000 emails in the total email archive corpus, which is far too many to individually annotate. By restricting emails from the dev mailing list after the introduction of JIRA and removing emails with unreadable dates or attachments, we reduced this figure to 209572 emails over 2011-11-15 to 2017-04-27. To down-select further, we imposed the following traits as requirements:

- A distribution of email traces that contains variable lengths
- Emails should occur within a contiguous time block (not a sampling)
- Emails should contribute to both partial and complete workflow instances
- Total email volume must be manageable for human labelling

We performed some preliminary analyses to help down-select to a more reasonably sized, but representative time slice within the data. We used JIRA issues (bug reports and feature requests) as a guide to help assess the trace duration and trace lengths that exist in the data. Each JIRA issue is assigned a unique number for identification (JIRA ID) and most, but not all, JIRA actions result in the automatic generation of at least one email that is sent to one of the other mailing lists associated with the project. These automatic emails contain the JIRA ID and a label that defines the workflow action being executed, making it straightforward to generate rough estimates of trace lengths and durations.

**Fig 1:**
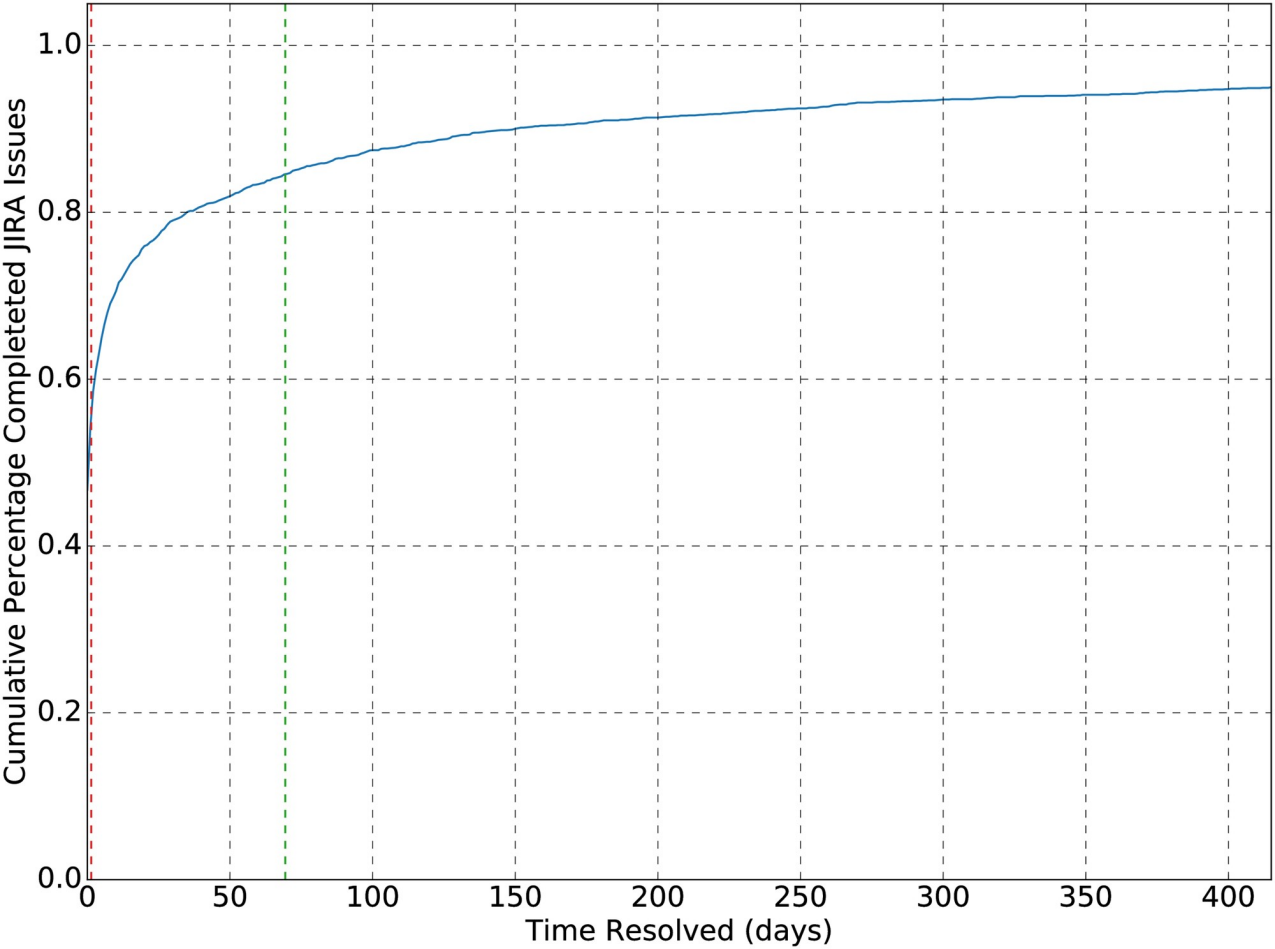
Time dependence of completed JIRA issues. 95% cumulative percentage of completed JIRA issues based on the time difference between creation and resolution of the issue. A mean time difference of 70 days, and a median of 1.3 days was observed.

**Fig 2:**
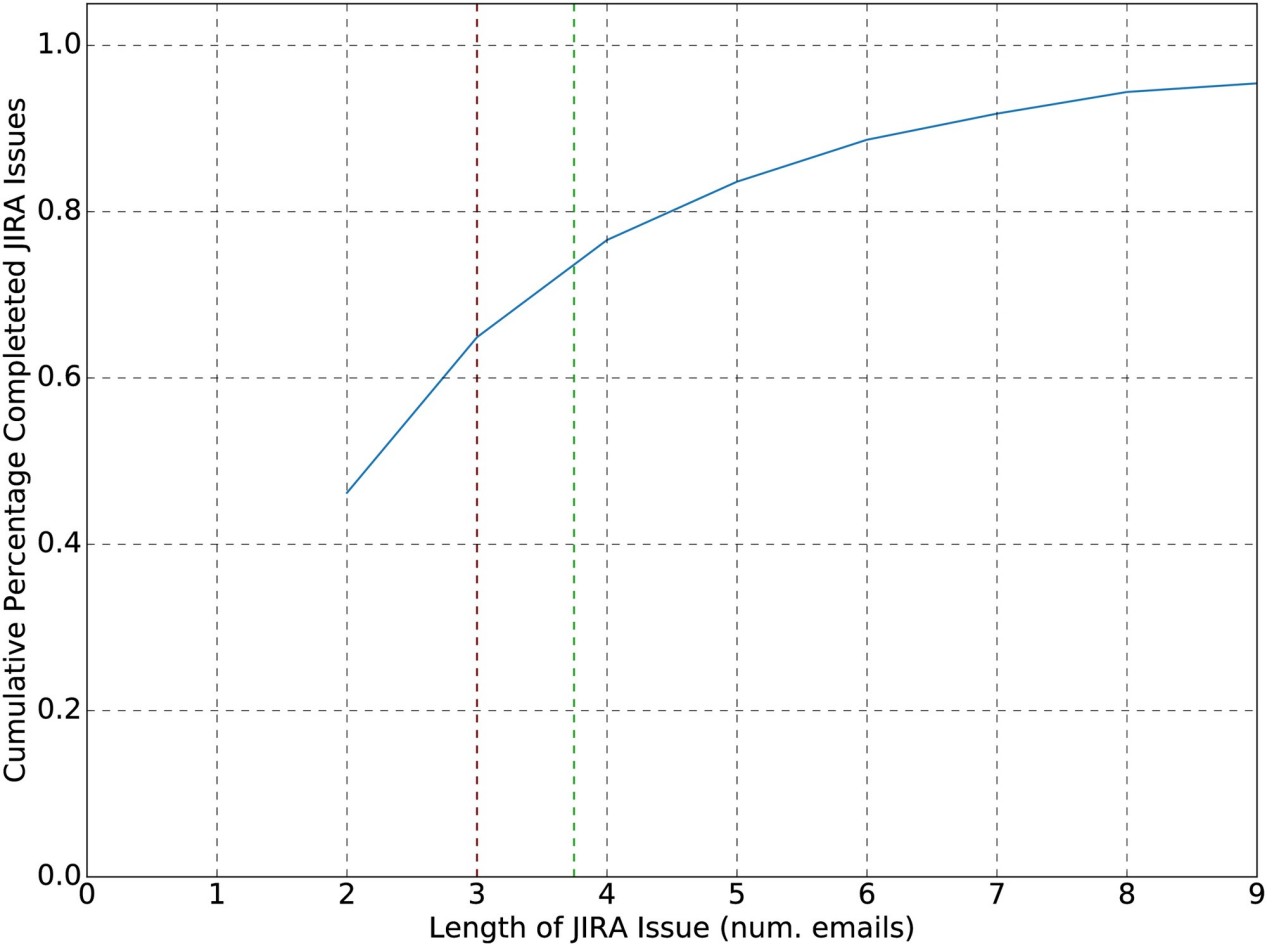
Length dependance of completed JIRA issues. 95% cumulative percentage of completed JIRA issues based on the length of their email chains from creation to resolution of the issue. A mean email length of 3.8, and a median of 3 was observed.

The six-year subset of data contains traces that follow both incomplete and complete JIRA issue resolution workflow instances. Within this subset, 55% of the total of 6825 JIRA issues contained traces that were marked as resolved, indicating (potentially) completed workflow instances. Of these resolved issues, approximately 50% were created and resolved within one day, with a mean time of 70 days and a median of 1.3 days, shown in Fig 1 as red and green, respectively. This implies that using a period of approximately a week should provide a high number of resolved JIRA issues, and subsequently a high number of traces and workflows to ground truth. With regard to trace length, approximately 50% of the resolved issues contained traces that were resolved within 2 emails, and 95% contained fewer than 9 emails; see Fig 2.

With this data in hand, we selected a 4 day time window containing a total of 250 emails, with 7 JIRA issues created and resolved within the time window and 12 partial JIRA issues of length 3 or more. This window also occurred shortly before a planned software release, ensuring that it contained a relatively higher density of resolved JIRA issues, over the period 2017-04-14 10:42:39 UTC to 2017-04-19 13:27:37 UTC; see http://mail-archives.apache.org/mod_mbox/camel-dev/201704.mbox/browser. This provided us an effective set of emails for us to manually annotate in order to produce more meaningful traces and workflows than just basic JIRA ID assignment.

## 4 Ground truth construction

Here we describe the methods by which we extracted “ground truth” information from the 250 emails, along with examples from the dataset. Our analysis team consisted of 8 individuals from the Australian Defence Science and Technology Group (DST Group), the US Naval Research Lab (NRL), and MIT Lincoln Laboratory (MITLL).

### 4.1 Methodology

Each member of the team was given the same set of 250 emails that contained the date, from, to, subject and body fields of an email. An exemplar of the email format is given below:

Date: [Day], [Date] [Time] [Time Zone]

From: [SENDER] <[SENDER]@[DOMAIN]>

To: [Mailing List]@camel.apache.org

Subject: [Subject Line]

[Email Body]

We note that the emails were provided without preprocessing for spelling or grammar corrections, to account for natural sources of “noise” that may affect labeling fidelity in future machine learning applications.

#### 4.1.1 Criteria for extracting labels and traces from natural language events

The ground truth exercise began by abstractly considering the following questions:

1. What are the activity(s) discussed or observed in the email based on its subject line and content body?
2. What are the main keywords in this email that helped you decide on the activity(s) observed in Question 1?
3. To which trace instance does this email belong? (Note that emails of reply chains may not necessarily be part of the same trace given that some messages may go off-topic or switch to a different task discussion altogether.)
4. What overall tasks are the traces trying to fulfill?

With these questions in mind, we performed a pilot study on a 50-email dataset from a slightly different part of the Apache Camel corpus. That exercise, which we do not report here, guided us to impose the following constraints on our results:

1. Every email must have at least one label.
2. Email labels must be present in the email body or subject line.
3. Obvious ticket numbers or sender/recipient information should be excluded from the email labels.
4. Every email belongs to at least one activity trace, even if it is a trace by itself.
5. Each metalabel should be a brief phrase, and each email should have one metalabel.
6. Each email should appear in a trace exactly once; emails cannot represent multiple steps in a trace.
7. Every trace is one-dimensional; branching in traces is not allowed.

#### 4.1.2 The Delphi process

An important aspect in building this ground truth is to ensure that workflows that are identified are as sound and complete as practicable. To build confidence in the accuracy of our ground truth, the analysis team met at regular intervals during its construction. At each meeting we used a consensus building method, known as the Delphi process [20], to come to an agreement on keywords, metalabels, and activity traces. We conducted several additional meetings to decide on action assignments, workflow instances, and to identify the representative workflows. The Delphi process consists of (1) forming a panel of people who construct questions, (2) individual construction of responses to the questions, (3) collecting the responses to determine a majority, and (4) a discussion and voting process to resolve any objections to the majority vote.

#### 4.1.3 Building consensus

We used a modified Delphi process tailored to satisfy our application of ground truth construction. Each member labeled email events from the set of 250 in three subsets: emails (0,50), (51,100), and (101, 250). After individually analyzing each subset, the panel convened for the Delphi method. Between each Delphi meeting we used a script to gather the responses of each panel member, normalize the keywords to make them case-insensitive and reconcile duplicates from plurals, and count the numbers of occurrences of each label, as described in Alg 3. The summarized list of labels with anonymized vote counts were then sent to each of the panel members before each Delphi meeting to help resolve ties and to allow the panel members to prepare an argument to advocate for keyword labels that were, in their opinion, underrepresented. Each member of the panel conducted the following:

1. Read the email’s subject and body:
  - Assign up to 5 key word labels and 1 metalabel (defined in 2) that best summarize/describe the activities.
  - Assign this email to a trace based on its context.
2. Add all responses to a pool forming a majority opinion/decision on each event.
3. Receive pooled opinions and prepare argument if desired.
4. Convene with the panel to discuss as a group and each person has the opportunity to argue their justification, with the final result being determined by preferential voting.

An example of this process for keyword labels is provided in Table 1. In this example, four panel members submitted five keywords each to make a combined total of six keyword labels. Labels *C* through *F* received unanimous votes, and label *A* received 3 votes. At the Delphi meeting, the presumed resolution would be to select labels *A* and *C* through *F* unless any member wished to strongly advocate for label *B* or noticed deficiencies in the set of the presumed winners. The data presented to the panel members did not show the middle columns of Table 1; it only provided the labels and the total counts (the first and last columns). Occasionally, panel members voted to reconsider formerly resolved email labels to ensure labeling consistency in the current and later meetings.

**Table 1:** Example instance of keyword label consolidation by the Delphi leader before instituting the Delphi process.

Email Label	Panel Member 1	P.M. 2	P.M. 3	P.M. 4	Total Counts
Label *A*	1 vote	1	1	0	**3**
Label *B*	0 vote	0	0	1	1
Label *C*	1 vote	1	1	1	**4**
Label *D*	1 vote	1	1	1	**4**
Label *E*	1 vote	1	1	1	**4**
Label *F*	1 vote	1	1	1	**4**

To assist in the reproducibility of this work and better explicate our approach, we have broken down the process into a series of algorithms. Alg 1 summarizes the entire procedure that each member of the team underwent to arrive at consensus keyword, metalabel, traces, actions, and workflows. It makes references to two other sub-algorithms: DoDelphiKeywords, described in Alg 2 and DoWorkflowLabeling, described in Alg 4. We have attempted to indicate where a team member is expected to perform a decision with the comment “Individual chooses” where appropriate. In Alg 1 this occurs at the points at which keywords, metalabels, and traces are assigned.

**Algorithm 1**: **The overall ground truth algorithm used to establish labels, metalabels, traces, and workflows**

**Data:** Email dataset: Emails[250]

**Result:** Labels, Metalabels, Traces

*K* ← [] /* Keyword labels for each email              */

*M* ← [] /* Metalabels for each email                */

*T* ← [] /* Traces of emails                    */

**for** *i* ← 1 **to** 250 **do**

 *e* ← Emails[*i*]

 *K*[*e*] ← 〈*k*_1_^*e*^, …, *k*_5_^*e*^〉 /* Individual chooses            */

 *M*[*e*] ← *m*^*e*^ /* Individual chooses                */

 *τ* = FindAssociatedTrace(*e*, *K*[*e*], *M*[*e*], *T*) /* Individual chooses   */

 **if** *τ* ≠ ∅ **then**

  *τ* = *τ* ∪ *e*

 **else**

  *T* = *T* ∪ NewTrace(*e*)

 **end**

 **if** *i* ∈ {50, 100, 250} **then**

  *K* ← DoDelphiKeywords(*Emails*, *K*)

  *M* ← DoDelphiMetalabels(*Emails, K, M*)

  *T* ← DoDelphiTraces(*Emails, K, M, T*)

 **end**

**end**

Workflows ← DoWorkflowLabeling(*Emails*, *K*, *M*, *T*)

Alg 2, DoDelphiKeywords, represents an instance of the Delphi consensus building meetings that occurred throughout the exercise. Essentially, it shows how the Delphi leader assembled the labels from each panel member, how each panel member prepared for the meeting by observing the rankings, and how the meetings actually proceeded to arrive at the consensus keywords for each email. The process for deciding upon consensus metalabels, traces, action labels, and workflows was similar, but we do not provide explicit algorithms for the Delphi processes for each of those cases; the explicit example in Alg 2 can be considered as a template for the remaining data products. As an example, a candidate trace could contain 8 out of 10 emails in consensus before the Delphi meeting, so, during the meeting, we would debate and then vote to decide upon the allocation of the 2 remaining emails in conflict.

**Algorithm 2**: **DoDelphiKeywords**: **The Delphi process employed for consensus building in email keyword labeling at the 50, 100, and 250 emails**.

**Data:** N: either 50, 100, or 250

**Data:** K: set of email keyword labels and Emails

**Result:** FinalKeywords

E ← N-50

**if** *N* == 250 **then**

 E ← 100

**end**

KeywordDictionaries ← SummarizeKeywordLabels(*K*, *E*, *N*)

*arguments* ← []

**for** *p* ← 1 **to** |*Panel_Members*| **do**

 *arguments*[*p*] ← []

 **for** *i* ← *E* **to** *N* **do**

  Top5Keywords ← SortByDescendingVotes (KeywordDictionaries[i])

  **if** *p disagrees with Top5Keywords* **then**

   *arguments*[*p*][*i*] ← Prepare argument for disputed labels in email *i*.

  **end**

 **end**

**end**

**for** *i* ← *E* **to** *N* **do**

 Top5Keywords ← SortByDescendingVotes (KeywordDictionaries[i])

 **for** *p* ← 1 **to** |*Panel_Members*| **do**

  **if** *arguments*[*p*][*i*] ≠ ∅ **then**

   Present *arguments*[*p*][*i*] to rearrange the top 5 keywords

   Top5Keywords ← keywords with new voting results

  **end**

 **end**

 FinalKeywords[i] = Top5Keywords

**end**

**return** *FinalKeywords*

Alg 2 makes reference to another algorithm, shown as Alg 3, in which the Delphi leader normalizes the labels and anonymizes the votes to summarize the results before the consensus meeting that occurs at the end of Alg 2. The embedded TextNormalize function can be thought of as a data cleansing step to ensure that the final labels are case-insensitive and stemmed, preventing scenarios like “commit”, “Commit”, and “committing” from being considered as unique keyword labels for a particular email.

**Algorithm 3**: **SummarizeKeywordLabels**: **The process to normalize and aggregate all of the panel members’ submitted keyword files**.

**Data:** N, either 50, 100, or 250

**Data:** Labels.csv from each panel member

**Result:** KeywordDictionaries

E ← N-50

**if** *N* == 250 **then**

 E ←100

**end**

KeywordDictionaries ← ∅

**for** *file in panel_member_submissions* **do**

 **for** *i* ← *E* **to** *N* **do**

  **for** *k in K*_*i*_ **do**

   *k* ← TextNormalize(*k*)

   **if** *k in KeywordDictionaries[i]* **then**

    KeywordDictionaries[*i*][*k*] += 1

   **else**

    KeywordDictionaries[*i*][*k*] ← 1

   **end**

  **end**

 **end**

**end**

**return** *KeywordDictionaries*

At the conclusion of the meetings to resolve the email labels, metalabels, and trace allocations, the panel members allocated the traces to workflows as described in Section 5, the DoWorkflowLabeling algorithm described in Alg 4. Essentially, given a collection of emails, keywords, metalabels, and traces, this algorithm describes how each panel member chose “actions” to assign to each email event and to then look for patterns in the trace instances to construct workflows. The consensus metalabels provided a good guide in the selection of actions; frequently, the action labels were a coarser instance of the metalabels. In addition to allocating traces to workflows by looking for pattern recognition, we also counted the numbers of instances of each edge occurring in the workflow graph as a stand-in for the support of each edge.

**Algorithm 4**: **DoWorkflowLabeling**: **High level description of the trace-to-workflow assignment algorithm**.

**Data:** *M* email metalabels

**Data:** *T* identified event traces

**Result:** Actions, Workflows

*W* ← ∅

*A* ← ∅

**for** *τ in T* **do**

 *a*_*prev*_ = ∅

 **for** *e in τ* **do**

  *a* = FindAssociatedAction(*e*, *M*[*e*], *τ*, *A*)

  **if** *a* = ∅ **then**

   *a* = NewAction(“action_label”)

   *A* = *A* ∪ *a*

  **end**

  *w* = FindAssociatedWorkflow(*a*, *W*)

  **if** *w* = ∅ **then**

   *w* = NewWorkflow ()

   *W* = *W* ∪ *w*

  **end**

  *w*_*A*_ = *w*_*A*_ ∪ *a*

  **if** *a*_*prev*_ ≠ ∅ **then**

   *w*_*D*_ = *w*_*D*_ ∪ (*a*_*prev*_, *a*)

  **end**

  *a*_*prev*_ = *a*

 **end**

**end**

(*A*, *W*) ← DoDelphiWorkflows(*A*, *W*)

## 5 Results

In this section, we provide samples of the consensus results produced during the Delphi process; links to the full results are provided in Section 8. We note that we disallowed the use of JIRA issue numbers as valid consensus keywords early in the Delphi meetings, although those labels do appear in the individual votes.

Keyword labeling results for emails 102-107 are shown in Table 2. Each email has an email ID ordered temporally, followed by a list of the 5 resulting keywords and the number of votes received for each keyword label. The consensus keywords are not always English language words.

**Table 2:** Consensus of keyword labels for emails 102-107 following the Delphi process meetings.

Email ID: 102	Email ID: 103	Email ID: 104
(‘created’, 6)	(‘docker’, 8)	(‘commented’, 7)
(‘doc’, 5)	(‘upgrade’, 7)	(‘release’, 6)
(‘ascii’, 5)	(‘java’, 7)	(‘accessexternaldtd’, 5)
(‘task’, 4)	(‘commit’, 6)	(‘xerces’, 4)
(‘component’, 4)	(‘version’, 6)	(‘property’, 2)
Email ID: 105	Email ID: 106	Email ID: 107
(‘pom.xml’, 6)	(‘doc’, 6)	(‘resolved’, 7)
(‘camel-catalog’, 6)	(‘ascii’, 6)	(‘ascii’, 6)
(‘fix’, 6)	(‘commit’, 5)	(‘doc’, 5)
(‘javadoc’, 5)	(‘component’, 4)	(‘component’, 4)
(‘commit’, 5)	(‘warn’, 1)	(‘fixed’, 3)

The metalabel results for emails 102-107 are shown in Table 3. One of the main reasons for metalabels is to succinctly describe the event represented in the underlying email. This provides a first layer of abstraction that helps align the emails with the traces and to later assist in the assignment of action labels.

**Table 3:** Consensus meta-labels for emails 102-107 following the Delphi process meetings.

Email ID	Meta-labels
102	task creation
103	commit code modification
104	bug issue comment
105	commit fix
106	commit fix
107	task issue resolved

Using the keywords and metalabels, the team derived 65 traces from the 250 emails. Four example consensus traces are provided in Table 4. Trace 30 assigns three of the emails (100, 101, and 104) depicted in Tables 2 and 3 to a single trace associated with activities that occur in the middle of an issue workflow instance. Similarly, trace 31 assigns three of the emails (102, 106, and 107) to a single trace associated with the creation, code commit, and closing of a JIRA issue. The other two traces only contain single events.

**Table 4:** Consensus trace descriptions for traces 30-33.

Trace ID	Summary description	Email IDs
30	CAMEL-11000 property accessexternaldtd not recognized	100, 101, 104
31	CAMEL-11160: Component docs—ASCII doc warns	102, 106, 107
32	upgrade Docker java version 3.0.9	103
33	fix pom.xml camel-catalog javadoc generated	105

These traces, keywords, and metalabels guided the extraction of 19 actions shown in Table 5 that each describe a significant task in the email chain. To evaluate the quality and validity of these actions, we inspected the underlying keywords associated with these actions. We combined the 5 keywords for each email associated with a particular action to create a larger keyword-action dataset. We then used this to measure the frequency distribution of these words for a given action. The quality and uniqueness of an action could thus be compared by measuring the sharpness of each frequency distribution and its overlap with other action-keyword frequency distributions. Given an action-keyword frequency distribution and its mean keyword, the overlap with all other observed action-keyword frequency distributions within 1 standard deviation of the mean is denoted OL(*σ*), within 2 standard deviations OL(2*σ*), and for all the keywords within the distribution, OL(all). Therefore, a standard deviation value of *σ* = *x* with *OL*(*σ*) = *y* would imply that there were *x* unique keywords within 1 standard deviation of the action-keyword frequency distribution’s most observed keyword, and the fraction *y* of these *x* unique keywords were observed in other action-keyword frequency distributions. These results are summarized in Table 5.

**Table 5:** Actions, and their observed frequencies, constructed from the traces, keywords, and metalabels during the Delphi process. *σ* denotes the standard deviation of the an action-keyword frequency distribution, and OL(*σ*) the fractional overlap with other action-keyword frequency distributions within 1*σ*.

Action	Frequency	*σ*	OL(*σ*)	OL(2*σ*)	OL(all)
commit changes	87	24.67	0.36	0.49	0.49
issue comment	23	13.43	0.67	0.87	0.90
ask a question	18	36.38	0.41	0.57	0.59
create issue	17	16.97	0.36	0.56	0.60
issue update	15	11.15	0.64	0.85	0.91
provide support	13	35.33	0.51	0.69	0.69
resolve issue	12	11.92	0.62	0.85	0.90
close pull request	11	16.51	0.67	0.91	0.96
automated comment issue	10	6.10	0.64	0.90	0.96
build system update	10	2.10	0.20	0.24	0.24
assign issue	9	14.69	0.31	0.49	0.56
open pull request	8	12.28	0.65	0.93	0.95
update question	4	10.64	0.55	0.70	0.70
version release planning	4	8.64	0.40	0.60	0.65
close question	3	10.23	0.53	0.80	0.87
reopen issue	2	3.82	0.40	0.70	0.80
work started issue	2	6.82	0.26	0.42	0.42
distribute situational awareness	1	3.41	0.0	0.0	0.0
publicity	1	3.41	0.0	0.0	0.0

Table 6 provides the action labels for traces 30–33. These can be compared with the keywords and metalabels for their constituent emails provided in Tables 2 and 3.

**Table 6:** Consensus action descriptions for traces 30-33.

Trace ID	Action List	Email IDs
30	issue comment, issue update, issue comment	100, 101, 104
31	create issue, commit changes, resolve issue	102, 106, 107
32	commit changes	103
33	commit changes	105

**Fig 3:**
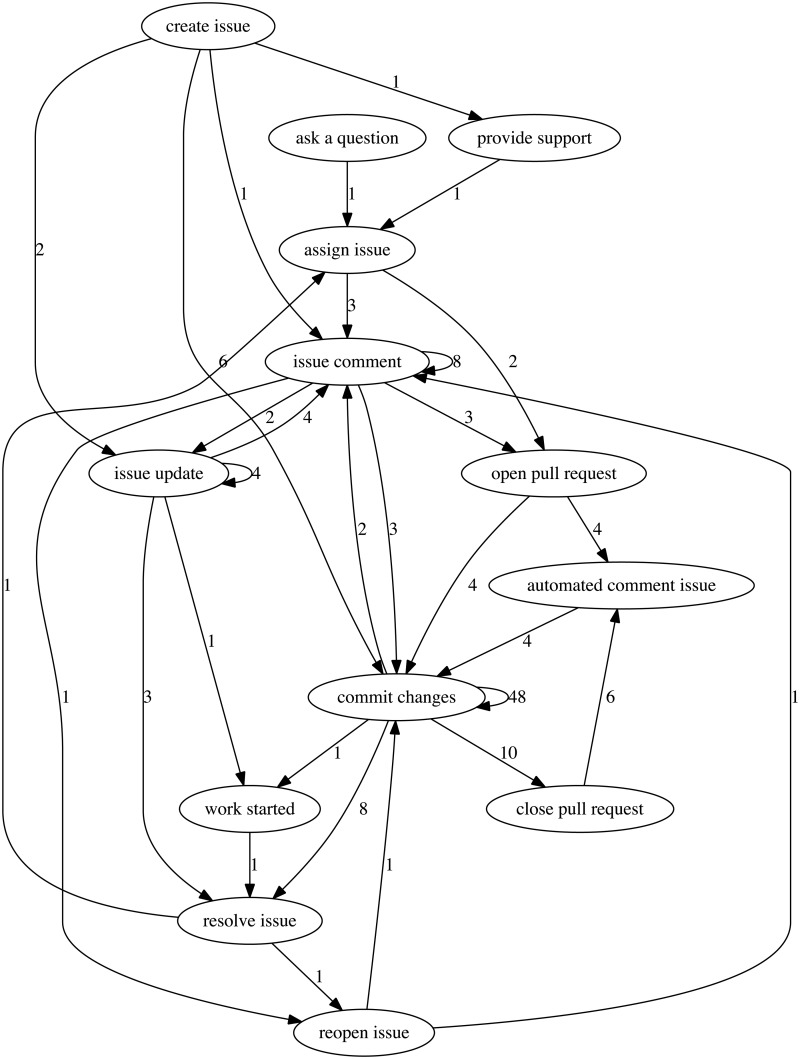
Workflow digraph containing the actions relating to a “bugfix”. Numbers between each directed edge (including self referential connections) indicate the number of instances of this connection.

**Fig 4:**
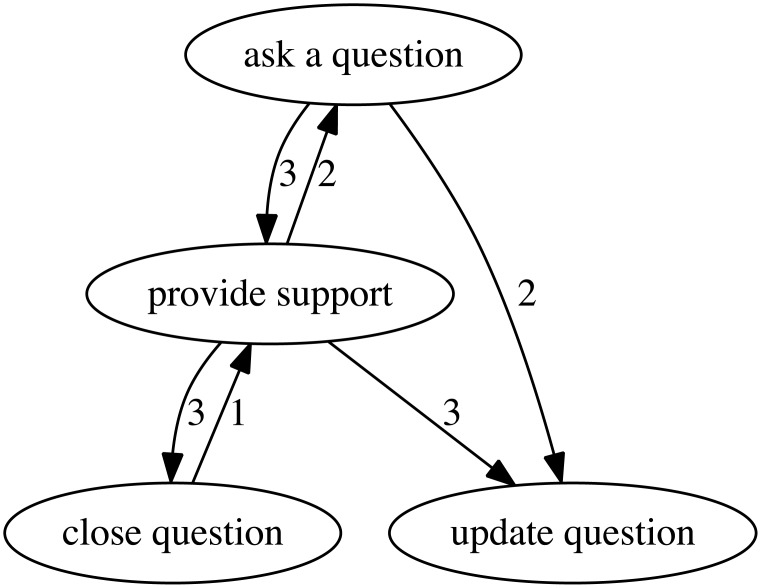
Workflow digraph containing the actions relating to “support”. Numbers between each directed edge (including self referential connections) indicate the number of instances of this connection.

The final workflows were constructed from the consensus actions, where the actions and sequencing in the workflow instances were denoted as digraph vertices and edges, respectively. This resulted in 6 consensus workflow labels, summarized in Table 7. Four of the workflows were trivial, containing only one action with self referential loops. Two non-trivial workflows are shown in Figs 3 and 4. The complete allocation of traces, action labels, and workflow labels is provided in the S5 File (8).

**Table 7:** Workflow labels and incidence. Note that they do not sum to 65 since two workflow instances overlap as the “bugfix” and “user support” workflows.

Workflow	Count
bugfix	47
user support	14
system update	3
situational awareness	1
release planning	1
publicity	1

## 6 Discussion

90% of the observed workflow instances in the dataset mapped to the dominant two workflows, *support* and *bugfix*. The fidelity of these workflows were heavily dependent on the granularity of the chosen actions. There is a tension between over-generalizing actions versus assigning actions that are too specific. The former may result in a single digraph that encapsulates all workflow instances, whereas the latter could result in an excessive number of overly specific digraphs. This drove us to a consensus selection of 19 actions unique enough to differentiate workflow instances whilst still remaining general enough to construct meaningful workflow models. For example: the *bugfix* digraph contains workflow instances that result in creation, modification, and completion of code, regardless of the specific type (e.g. feature additions, improvements, or bugs); the *support* digraph contains workflow instances that result in user and developer questions, support, and updates; the *build system update* digraph contains workflow instances that result in updates to the Camel servers; and so on. The result was the identification of different processes occurring in the overall Camel system.

Several discussions revolved heavily on the assignment of several actions that were deemed too similar by certain members of the panel (e.g. “issue comment” vs. “issue update”, and “update a question” vs. “asking a question”). The actions which were more heavily discussed are measurably different from other actions in terms of their overlap. Actions such as “build system update”, “assign issue”, and “commit changes” had less than 35% overlap within 1 standard deviation of their most frequent word, and 50% overlap within 2 standard deviations in comparison with other actions; this may indicate that these actions were more uniquely defined. On the other hand, actions such as “issue comment” and “issue update” had closer to 65% and 85% overlap, respectively. Generally, the overlap values indicate that the peaks of the action-keyword frequency distributions are differentiable, and thus we believe the actions are an accurate representation of the tasks within the workflow instances. Further investigation is needed to determine whether taking samples from different subsets of the larger Camel dataset results in more workflow instances and subsequently a greater distribution of actions to be observed, thereby improving the accuracy of our chosen actions. In the supplementary information for the consensus keyword labels, we are also providing each panel member’s vote for each keyword label as another means of assessing the “strength” of the keyword-to-email (and to-action) assignment. For instance, some emails had strong keyword-label consensus before the Delphi meetings, so it may be more likely for those keywords to be identified and associated to actions, whereas emails/actions with very weak keyword-label consensus may correspond with broader overlaps.

As described in Section 2, it is possible to extract a workflow using partial workflow instances if enough of these partial workflow instances exist in the data. For example, trace 30 and 31 in Table 6 were initially mapped to two workflow instances that were assigned to two separate digraphs because there was no overlap in their actions. However the existence of other workflow instances eventually created a connection between actions of these workflows resulting in their consolidation to the same digraph (e.g. bugfix). We did observe that some digraph linkages only had a small number of directed edge counts to link together digraphs. For example, the digraphs for “bugfix” and “support” have a connection between the graph vertices “provide support” and “ask a question”, but we were uncertain as to whether this connection was an isolated instance or generalizable as part of the workflow. In order to maintain the generalizability of the digraphs, we decided that vertices with two or less edge counts did not have enough support and were omitted from the final result. Further work may consider increasing the size of the considered dataset, which would be required to further investigate whether these are meaningful connections in producing workflows.

This dataset yields several advantages for initial research into automated workflow analysis. For instance, several of the traces were readily tagged with JIRA tracking (ticket) numbers, allowing for easy identification of connected events. Also, the workflows that correspond to these issues typically have regular observable beginnings and endings (opening and closing the tickets) which make these particular emails, traces, and workflows valuable for analysis, since the JIRA server mediates many of the workflows performed by the developers and users.

However, these advantages come at the cost of a potential loss of generality. Clearly, if one is attempting to correlate traces with their underlying workflows in the absence of a regular, machine-driven mediator, these advantages will not be transferrable since there will be more variance in the human-driven processes. Therefore, successful resolution of this dataset with a trial algorithm alone is likely a necessity, but insufficient condition for application of more general problems. Although this dataset has sufficient complexity to challenge the algorithm designer, there are a number of approaches that one could employ to test the robustness of a trial workflow analysis algorithm. For instance, one could scramble the temporal ordering of the emails (fuzzing their times), add or remove random words from the emails, deliberately misspell certain words, drop time intervals to simulate sensor downtime, etc., in order to test the *robustness* of the trial algorithm. By parameterizing such noise, one could also calculate the sensitivities of trial algorithms to these data distortions.

Future work to improve this dataset may include enrichment of labels with the extant social network information arising from the cyber-personas (email addresses and observed names). For instance, as a first step, incorporating the from- and to-addresses in the emails would likely enhance the accuracy of a trace recognition algorithm. Another avenue might be to look for patterns of particular users in beginning and/or ending traces; users’ participation in a particular trace may be strongly indicative of a trace’s workflow membership. However, this dataset is deficient in varied cyber-personas (users) throughout the 250 emails, so it is possible that this data enrichment may not be practical. Many of the emails are sent to or received from large, group email addresses, such as jira@apache.org and issues@camel.apache.org, which dilutes this information.

It is worth noting that the Delphi consensus discussions often took longer than we expected. The total work effort was approximately 60 work hours per person (40 hours to analyze 250 emails, 10 hours to conduct the Delphi meetings, and 10 more hours for workflow analysis and the final Delphi meeting). For those who wish to attempt an extension of this work on the same dataset—or on a different dataset altogether—we recommend conducting the first Delphi meeting relatively early in the process (or a pilot run) to establish annotation requirements early on. As the meetings progressed, the consensus meetings ran more fluidly, but it was critical to engage in discussions about which and what combinations of keywords were permissible, etc., in order to truly achieve consensus. For instance, we decided during an early Delphi meeting to disallow JIRA issue numbers as valid consensus keyword labels to enhance the human-interpretable value of the keyword data, although the raw data does reflect some votes for those labels.

## 7 Related work

The driving component of our work is the combination of event classification and process mining techniques employed to perform workflow analysis. Existing research in both NLP and process mining fields discuss the use of labeled ground truth datasets to validate their approaches. However, we have yet to find a single labeled, ground truth dataset that can be used to validate the entire workflow analysis pipeline.

Email-activity management systems frequently use text classification techniques in supervised learning environments to label and classify activities. For instance, Drezde et al. [21] use the Naïve Bayes classifier and a set of labeled emails as a baseline approach to define email activity membership in their study. This type of classification technique is useful for static sets of labels, but does not accommodate well for dynamic sets of labels where new activity labels are continually added over time. Kiritchenko and Matwin [22] attempt to overcome this manual labeling problem by employing an approach called Co-Training, which requires only a small set of labeled examples and redundant features to produce initial weak classifiers; these classifiers will iteratively train each other on unlabeled data to improve their accuracy and performance. These types of text classification processes require large amounts of labeled training data to increase the accuracy of the classifier, however the workflows within these datasets are given by the process structure of the reply email chains, based on subject line and sender. The complex workflows that can be generated from the actions events by considering multiple email chains are absent from the manually annotated datasets these studies use. Thus, although the labeled datasets can be used to test the accuracy of keyword and metalabel generation, it is insufficient for workflow analysis.

Kalia et al. [23] focus their work on classifying activities based on commitment-based service engagements from chat and email messages. The goal of their study is to follow the progression of service commitments and capture the business relationships that are involved between two entities. They apply natural language processing and machine learning to identify the creation, delegation, cancellation, and/or completion of a commitment. First, they group all emails replied to or forwarded with the same subject line into one conversation thread. Chats are, by default, grouped by thread. Second, in each conversation thread, they identify whether the message contains a task, or business activity, by using the typed-dependency method [24] to find relationships among words in each sentence. Finally, if the task indicates the creation of a commitment they use key features (e.g., present or past tense, action verbs, and negative verbs) to identify the stages of that commitment within each conversation thread. Thus, they are able to group commitments based on particular tasks. This approach takes event classification and discovery one step further by including event sequencing through the discovery of service commitment progression workflows. Their work used an automatic labeler to extract events related to the commitment workflow from the data, then had two annotators manually group these events into actions of creation, delegation, cancellation, and/or completion. Although the complexity for the commitment workflow would have been sufficient for our work, we want to be able to extract any or all workflows from within a dataset, thus the single ground truth commitment workflow in this dataset is insufficient for workflow analysis.

Process Mining (or Workflow Mining) is a set of algorithms for discovering workflows in an organization based on labeled event logs [25], or PCAP data [26]. The general concept is to construct a complete business workflow using exemplar cases within an event log which represent instances of the workflow being followed. Each case may vary, and not reflect the entire workflow due to potential existence of conditional, alternative or optional steps, but the aggregate of all cases has been shown to produce a more accurate workflow than any individual sequences. Process mining has applications across many domains, especially in organizations whose operations are high tempo, naturally workflow-oriented, but requires fluidity due to dynamism (such as hospitals, logistics, media companies and crime investigation agencies). Process mining is designed to augment workflow systems, as such it requires as a minimum labeled event sequences. This means that raw datasets (network traffic or user event logs) cannot be studied without having already gone through event classification and sequencing. Work in [27, 28] uses the verbiage inherent in procedural texts to sequence tasks. Di Ciccio and Mecella describe a declarative approach for mining control-flow constraints between tasks; our intended application of this data is most similar to their approach [4]. Delicado et al. provide an NLP-based capability that links text-based descriptions of process models to a standard business process model description in their NLP4BPM tool [6]. In a discussion of challenges and opportunities of applying NLP to business process management, van der Aa et al. similarly focus on the potential utility of NLP in alleviating the burden of elucidating a process from its text description or comparing between processes [29]. However, we found no datasets within process mining that contained labeled, sequenced natural language events for the purposes of discovering business process models from raw text.

Other, less standard, ground truthed datasets were also considered, however they often had a lack of data availability or accessibility to us. For instance, the 4TU Datacentrum hospital logs [30] contain workflows for patient treatment and movement within a hospital, however, all natural language events were in Dutch and generally very short.

## 8 Conclusion

In this work we constructed a ground truth dataset that describes a subset of the business functions for an open source software project in order to facilitate methods for automated workflow analysis. This ground truth contains manually annotated keywords, metalabels, traces, and actions, via the Delphi consensus method, that serve as meaningful descriptors to construct the workflows that best describe these business functions. This provides the missing link between semantic descriptions of mission goals and observable events in cyberspace, enabling researchers to quantify the efficacy of automated algorithms for workflow discovery analysis, as well as other automated analysis techniques within the fields of natural language processing and business process mining. This dataset enables future researchers in the area of workflow analysis to understand the methodology that was used to establish the ground truth, including our novel presentation of mapping sequences of natural language events to workflows. We also provide a way to replicate the approach using an extension of our source data or any other source data and provide a foundation for the testing and constructing of automated workflow analysis techniques. The Delphi consensus method results, labeled data, and final workflow results are available through the links in Section 8.

## Supporting information

S1 File Consensus keyword labels. This comma-separated-value (CSV) file contains the consensus keyword labels for each of the 250 emails, indexed by local email ID. It also contains the *j*th Delphi member’s individual vote for keyword *k* under the heading “Keyword_*k*_member_*j*”.
(CSV)

S2 File Consensus metalabels. This CSV file contains the consensus metalabels for each of the 250 emails, indexed by local email ID.
(CSV)

S3 File Consensus traces. This CSV file contains the 65 consensus traces for each of the 250 emails. Each trace is indicated by a sequence of email IDs.
(CSV)

S4 File Consensus workflow and action labels. This CSV file corresponds with the traces file in the S4 File (8), but the traces are assigned to workflow labels and the email IDs are replaced with consensus action labels.
(CSV)

S5 File Workflow construction data, CSV format. This CSV correlates the email IDs, times, trace IDs, and actions to enable the construction of workflows. It can, for instance, be used as an input file to bupaR, “Business Process Analysis in R”, available at https://www.bupar.net/.
(CSV)

S6 File Workflow construction data, XES format. Upon reviewer request, the workflow construction data in 8 is also provided in the eXtensible Event Stream (XES) format defined at http://xes-standard.org.
(XES)
